# Identifying Risk and Protective Factors in Recidivist Juvenile Offenders: A Decision Tree Approach

**DOI:** 10.1371/journal.pone.0160423

**Published:** 2016-09-09

**Authors:** Elena Ortega-Campos, Juan García-García, Maria José Gil-Fenoy, Flor Zaldívar-Basurto

**Affiliations:** Standing Seminar on Juvenile Justice, Psychology Department, University of Almeria, Carretera de Sacramento s/n. C.P. 04120, La Cañada de San Urbano, Almería, Spain; Universite de Bretagne Occidentale, FRANCE

## Abstract

Research on juvenile justice aims to identify profiles of risk and protective factors in juvenile offenders. This paper presents a study of profiles of risk factors that influence young offenders toward committing sanctionable antisocial behavior (S-ASB). Decision tree analysis is used as a multivariate approach to the phenomenon of repeated sanctionable antisocial behavior in juvenile offenders in Spain. The study sample was made up of the set of juveniles who were charged in a court case in the Juvenile Court of Almeria (Spain). The period of study of recidivism was two years from the baseline. The object of study is presented, through the implementation of a decision tree. Two profiles of risk and protective factors are found. Risk factors associated with higher rates of recidivism are antisocial peers, age at baseline S-ASB, problems in school and criminality in family members.

## Introduction

Antisocial behavior has no single definition, but it encompasses a variety of conducts and is influenced by the historical and social contexts wherein it takes place [1]. Sanctionable antisocial behavior (S-ASB) refers to those conducts that are registered as criminal or penal code violations in each country. S-ASBs represent one part of antisocial behavior [2].

Juvenile delinquency is currently one of the criminological problems that receive the most attention internationally [3–5]. Intervention with recidivist juveniles becomes essential in order to keep sanctionable antisocial behavior from persisting into adulthood, and keep juveniles from pursuing a chronic criminal course throughout their lifetime [6].

Research in juvenile justice underlies the “*What Works*?” approach [7,8], which seeks to identify what is effective in reducing S-ASBs in young offenders. Their experience with juvenile justice is to be as effective as possible and specific, appropriate resources are to be applied to each juvenile according to the S-ASB committed and to the juvenile’s psycho-socio-educational situation [9]. Research has shown that more restrictive sanctions are not more effective [10].

The theory of the Psychology of Criminal Conduct (PCC) [9] was created to offer concepts for better planning and execution of intervention with offenders. According to this theory, there are factors that increase committing an S-ASB, called Risk Factors [9]. A risk factor for S-ASB is a variable that predicts a high probability of recidivism [11,12]; by contrast, protective factors present a lower probability of recidivism, and mitigate the effect of risk factors [13,14].

The *Risk-Need-Responsivity* model (RNR) [15] presents eight factors related to committing an S-ASB. These factors’ strength of association with the behavior is not homogeneous; the authors group the eight factors into two groups according to their predictive strength. The factors most closely associated with S-ASB, known as the *big four*, are antisocial attitudes, antisocial peers, antisocial personality pattern and a history of antisocial behavior. The group that is more loosely associated with S-ASB, *the moderate four*, are the factors of family and/or partner, school and/or work, free time and leisure, and substance abuse. Factors included in the RNR Model have a dynamic nature, they are modifiable, except for the factor of prior criminal history [16]; while the latter is not a criminogenic need, it is included because of its close relationship to S-ASB [15].

Research on recidivism in juvenile justice has made great efforts to identify risk factors presented by young offenders, with the understanding that their elimination would reduce S-ASBs [10]. Research on risk and protection factors indicates that the factors most strongly associated with repeat S-ASBs are criminal history [17], age at the first S-ASB [18], problems at school or work [17–24]; antisocial peers [18,20,25–27]; poor use of leisure time [17,18,20,28,29]; antisocial personality/behavior [17,27]; lack of parental supervision [27,30] and criminality in family members [31,32].

This study has two objectives. The first objective is to estimate the percentage of recidivism of juvenile offenders that pass through Juvenile Justice. The second objective is to understand the profile of young offenders, paying attention to any differentiating characteristics in the group of repeat offenders.

## Method

### Participants

The data were extracted anonymously from the youth court records with the permision of the juvenile court of Almeriía. None of the researchers had access to the juveniles personal data. This study was approved by the Ethics Committee of the University of Almeria, and conducted in compliance with the Declaration of Helsinki and Spanish legislation on personal data protection.

The study sample was made up of the set of juveniles who were charged in a court case in the province of Almería (Spain) during a year of study. Their first case opened during the period of study is taken as the baseline incident. The juveniles included in this study had committed some S-ASB specified in the Spanish Penal Code. Any juvenile who commits an S-ASB will be judged under Organic Law 5/2000 if at the time of the act he/she was between the ages of 14 and 18. The sample was composed of a total of 594 juveniles.

### Variables

The data records of the youth court start with the information recorded from the moment when the young offender enters in the Juvenile Justice System. In that moment, the workers of the Juvenile Court (a psychologist, a social worker and an educator) interview them and made an evaluation to guide the judge in his decision. This evaluation is based on the protective and risk factors presented by the juvenile. During the data extraction phase, the researchers could ask to the Forensic Psychologist any doubts regarding the juvenile psychological evaluation.

The variables addressed in this study are classified into three groups, individual, criminological and contextual variables, according to the variable’s relation to the juvenile.

### Recidivism

For this study, there is recidivism of S-ASB when the juvenile is charged in a new court case in the Juvenile Court of Almeria (Spain) at some time after the baseline case established as point of reference. The recidivism study covered a period of two years from the date of each offender’s baseline court case [17,33,34].

### Data analyses

A decision tree was constructed for the total group of juveniles, using statistical software IBM SPSS Statistics 20.0. The objective of the decision tree was to create homogeneous groups based on the value of a resulting or dependent variable (in this study, recidivism of the S-ASB), grouping subjects into two or more groups as a function of the predictive variables under study [35,36]. The algorithm used was Exhaustive Chi-squared Automatic Interaction Detection, or exhaustive CHAID [37–39].

Classification methods such as the decision tree are very suitable for understanding the profiles of youth with high and low probabilities of delinquency [40–45]. One of the advantages of this analysis is the format of the output: a very intuitive, easily interpreted graphic, appropriate for contexts where application does not involve research experts, such as in the case of juvenile justice system personnel [46].

## Results

### Juvenile Offenders

The set of juveniles in this study primarily consists of male youths (85.4%) with Spanish nationality (79%). Of the juveniles included in this study, 57.4% were 14–15 years old at the time they committed their first S-ASB. At the time of the baseline incident, 54.9% were from 16 to 17 years old (Table 1).

**Table 1:** Frequency and percentage of the juvenile’s individual variables.

Characteristics	%(N)	%(N) Recidivism
Gender		
Male	85.4%(507)	90.5%(191)
Female	14.6%(87)	9.5%(20)
Nationality		
Spanish	79.0%(469)	80.6%(170)
Other	21.0%(125)	19.4%(41)
Age at 1st S-ASB		
14–15	57.4%(341)	69.7%(147)
16–17	42.6%(253)	30.3%(64)
Age at baseline S-ASB		
14–15	45.1%(268)	52.1%(110)
16–17	54.9%(326)	47.9%(101)
Repeated school year		
Yes	59.9%(356)	66.4%(140)
No	40.1%(238)	33.6%(71)
Dropout		
Yes	59.6%(354)	75.4%(159)
No	40.4%(240)	24.6%(52)
School absenteeism		
vYes	11.6%(69)	14.7%(31)
No	88.4%(525)	85.3%(180)
Interest in studies		
Yes	22.2%(132)	9.5%(20)
No	77.8%(462)	90.5%(191)
Organized leisure activities		
Yes	2.5%(15)	1.4%(3)
No	97.5%(579)	98.6%(208)
Substance abuse		
Yes	36.7%(218)	52.6%(111)
No	63.3%(376)	47.4%(100)
Physical abuse		
Yes	5.2%(31)	8.5%(18)
No	94.8%(563)	91.5%(193)
Psychological abuse		
Yes	3.4%(20)	4.3%(9)
No	96.6%(574)	95.7%(202)
Sexual abuse		
Yes	1%(6)	2.4%(5)
No	99%(588)	97.6%(206)
Physical health problems		
Yes	3%(18)	2.8%(6)
No	97%(576)	97.2%(205)
Mental health problems		
Yes	12.6%(75)	19%(40)
No	87.4%(519)	81%(171)
Partner		
Yes	10.6%(63)	9%(19)
No	89.4%(531)	91%(192)
Children		
Yes	2.4%(14)	1.4%(3)
No	97.6%(580)	98.6%(208)
Only child		
Yes	8.9%(53)	8.1%(17)
No	91.1%(541)	91.1%(194)
Violent		
Yes	9.1%(54)	12.8%(27)
No	90.9%(540)	87.2%(184)

Regarding the group of schooling-related variables, 59.9% of the juveniles had repeated at least one year in school, 11.6% showed absenteeism, and 59.6% had dropped out. Only 22.2% of the juveniles showed interest in their studies. Regarding the variables relating to the juvenile’s leisure, 97.5% of the juveniles did not participate in organized leisure activities and 36.7% presented substance abuse.

Regarding variables related to the juvenile’s physical and psychological well-being, more than 94% of the juveniles had not been a victim of physical, psychological or sexual abuse. 97% of the juveniles presented no physical health problems, and 87.4% presented no mental health problems.

As for the group of criminological variables that were studied (Table 2), 52.9% of the S-ASBs from the baseline court case were classified as misdemeanors, 59.8% were non-violent, with precautionary measures petitioned for 9.8% of the juveniles.

**Table 2:** Frequency and percentage of the juvenile’s criminological variables.

Characteristics	%(N)	%(N) Recidivism
Type of S-ASB		
Crime	47.1%(280)	53.1%(112)
Misdemeanor	52.9%(314)	46.9%(99)
Violence in the S-ASB		
With violence	40.2%(239)	39.8%(84)
Without violence	59.8%(355)	60.2%(127)
Educational measure imposed		
Measure imposed	52.2%(310)	63.5%(134)
No measure imposed	47.8%(284)	36.5%(77)
Accompanied in the S-ASB		
Accompanied	57.9%(344)	53.6%(113)
Alone	42.1%(250)	46.4%(98)
Victims in the S-ASB		
Yes	54.7%(325)	52.6%(111)
No	45.3%(269)	47.4%(100)
Interest in the proceedings		
Yes	86.4%(500)	78.7%(163)
No	13.6%(79)	21.3%(44)
S-ASB under the influence of substances		
Yes	1.5%(9)	0.9%(2)
No	98.5%(585)	99.1%(209)
Takes responsibility for the S-ASB		
Responsibility assumed	21.5%(128)	23.2%(49)
No responsibility assumed	78.5%(466)	76.8%(162)
Precautionary measures petitioned		
Yes	9.8%(58)	10.9%(23)
No	90.2%(536)	89.1%(188)

Regarding characteristics of the S-ASB committed, 57.9% were carried out in the company of others, 54.7% involved victims, and 98.5% of the juveniles indicated that they were not under the effects of substances at the time of the baseline incident. As for the consequences, 78.5% did not assume responsibility for the acts committed, 86.4% showed interest in the proceedings and 52.2% of the juveniles were assigned an educational measure.

Regarding contextual variables (Table 3), 39.7% of the juveniles revealed that their peer group of reference are antisocial peers. In the family setting, we note that 71.2% of the juveniles live with their parents, 51.5% have experienced changes in the home circle, and 91.1% have not witnessed unreported situations of violence in the family.

**Table 3:** Frequency and percentage of the juvenile’s contextual variables.

Characteristics	%(N)	%(N) Recidivism
Antisocial peers		
Yes	39.7%(236)	63%(133)
No	60.3%(358)	37%(78)
Unreported situations of violence		
Yes	8.9%(53)	13.7%(29)
No	91.1%(541)	86.3%(182)
Lives with nuclear family		
Yes	71.2%(423)	63%(133)
No	28.8%(171)	37%(78)
Ruptured family ties		
Yes	40.2%(239)	50.2%(106)
No	59.8%(355)	49.8%(105)
Traumatic disappearance of family member		
Yes	19%(113)	27%(57)
No	81%(481)	73%(154)
Changes in the home circle		
Yes	51.5%(306)	56.9%(120)
No	48.5%(288)	43.1%(91)
Paternal parenting style		
Adequate	37.9%(225)	19.4%(41)
No adequate	62.1%(369)	80.6%(170)
Maternal parenting style		
Adequate	34.5%(205)	15.6%(33)
No adequate	65.5%(389)	84.4%(178)
Father is violent		
Yes	10.6%(63)	15.2%(32)
No	89.4%(531)	84.8%(179)
Father’s nationality		
Spanish	77.1%(454)	77.6%(163)
Other	22.9%(135)	22.4%(47)
Mother’s nationality		
Spanish	78.1%(464)	78.7%(166)
Other	21.9%(130)	21.3%(45)
Father’s employment		
Active	78.3%(371)	70.3%(128)
Not active	21.7%(103)	29.7%(54)
Mother’s employment		
Active	66.7%(350)	67.8%(135)
Not active	33.3%(175)	32.2%(64)
Criminality in family members		
Yes	16.8%(100)	29.4%(62)
No	83.2%(494)	70.6%(149)
Physical health problems in family members		
Yes	1%(3.178)	16.1%(34)
No	86.9%(516)	83.9%(177)
Mental health problems in family members		
Yes	21.4%(127)	26.1%(55)
No	78.6%(467)	73.9%(156)
Father substance abuse		
Yes	14.3%(85)	20.9%(44)
No	85.7%(509)	79.1%(167)
Mother substance abuse		
Yes	4.9%(29)	6.6%(14)
No	95.1%(565)	93.4%(197)
Sibling substance abuse		
Yes	3.5%(21)	5.2%(11)
No	96.5%(573)	94.8%(200)

Regarding their parents, 37.9% of the fathers and 34.5% of the mothers present an adequate parenting style. More than 70% of the parents have Spanish nationality; 78.3% of the fathers and 66.7% of the mothers are employed.

Regarding the juvenile’s family members, physical health problems were not present in 86.9% of the cases, and 78.6% presented no mental health problems. The percentage of substance abuse was 14.3% in fathers, and less than 5% in mothers and siblings. Finally, 83.2% of the juveniles have no criminal record in the family.

### Recidivist Juveniles

The set of recidivist juveniles studied are largely male (95%) and have Spanish nationality (80.6%). At the time when they were first charged with S-ASB, 69.7% of the recidivist juveniles were 14–15 years old, while at the time of their baseline case, 52.1% of the recidivist juveniles were in this age range (Table 1).

Regarding variables related to educational context, 66.4% of the recidivist juveniles had repeated at least one school year, 14.7% showed absenteeism, and 75.4% had dropped out. Only 9.5% of the recidivist juveniles showed interest in their studies. In the area of leisure, 98.6% of the recidivist juveniles do not participate in organized leisure activities in their free time and 52.6% present substance abuse.

In the area of the juvenile’s physical and psychological well-being, 8.5% had suffered physical abuse, 4.3% were victims of psychological abuse and 2.4% of sexual abuse. Among the recidivists, 2.8% presented physical health problems, and 19% presented mental health problems.

As for the group of criminological variables, 53.1% of the S-ASBs committed were classified as crimes, 60.2% were non-violent, with precautionary measures petitioned in 10.9% of the cases. The juvenile who committed these acts primarily acted alone (53.6%) and there were victims in 52.6% of the cases. Considering the consequences of the behavior, 78.7% of the recidivist juveniles showed interest in the proceedings, 23.2% took responsibility, and 63.5% were assigned an educational measure (Table 2).

Regarding contextual variables (Table 3), 63% of the recidivist juveniles relate with antisocial peers and 13.7% have experienced unreported situations of violence. At the home life area, 63% live with their parents, 56.9% have experienced changes in the home circle and, in 50.2% of the families, some family tie has been broken.

As for variables pertaining to family members of the recidivist juvenile, 19.4% of the fathers and 15.6% of the mothers present an adequate parenting style, more than 75% of the parents are of Spanish nationality and 70.3% of the fathers and 67.8% of mothers are employed. In 29.4% of cases, there are family members with a criminal record. Physical health problems were present in 16.1% of the families of recidivist juveniles, and mental health problems in 26.1%. Regarding substance abuse, 20.9% of the fathers, 6.6% of mothers and 5.2% of siblings use substances.

### Decision tree study

**Fig 1:**
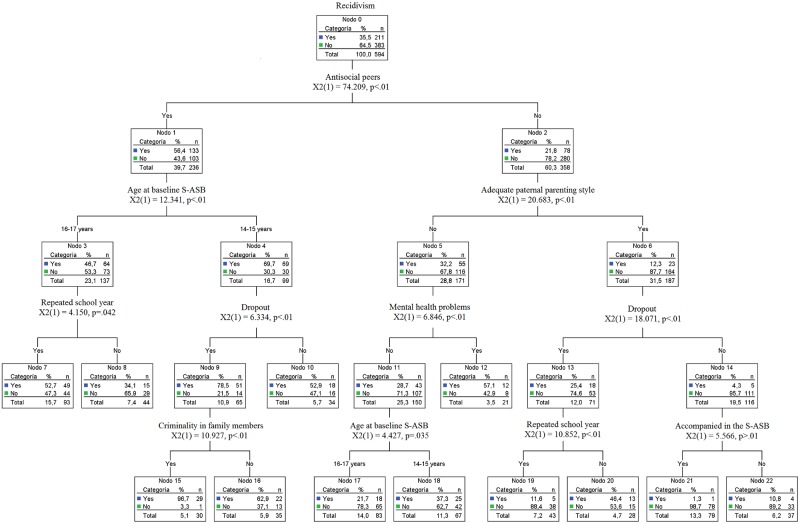
Decision tree.

The decision tree presents an estimation risk of 0.26 and a standard error of 0.01, correctly classifying 73.2% of the juveniles. The root variable in the decision tree is S-ASB recidivism; 64.5% of the juveniles included in this study were not recidivists. The variable that produces the first split in the decision tree is antisocial peers (χ^2^_(1)_ = 74.20; p < .01; d = 0.75). At this first divide, two branches appear in the decision tree: one branch, headed by the presence of *antisocial peers*, includes factors that encourage or are risk factors for S-ASB recidivism; another branch, headed by the absence of *antisocial peers*, includes factors that mitigate or protect against S-ASB recidivism in these young offenders, that is, factors that characterize the majority of the non-recidivists (Fig 1).

First, we describe the node where risk factors are concentrated. Juveniles characterized by having antisocial peers as their example show a percentage of recidivism of 56.4%. The variable that produces the next split in the decision tree is the age at baseline S-ASB (χ^2^_(1)_ = 12.34; p < .01; d = 0.47); juveniles whose first court case was opened at the age of 14–15 show a recidivism of 69.7%, while for the group whose first case was opened at age 16–17 this rate drops to 46.7%.

Proceeding to the next level of the tree, for the group whose first court case was opened at age 14–15, the variable that produces the next split is dropout (χ^2^_(1)_ = 6.33; p < .01; d = 0.53), the percentage of recidivism is 78.5% in the group of juveniles who have dropout. The variable that produces the next split is criminality in family members (χ^2^_(1)_ = 10.92; p < .01; d = 0.89). The percentage of recidivism is 96.7% in the group of juveniles who have family members with a criminal record, while for juveniles whose family members show no indications of criminality, the percentage of recidivism is 62.9%.

For the group of juveniles whose first court case was opened at age 16–17, the variable that produces the next node split is having repeated a year in school (χ^2^_(1)_ = 4.15; p < .05; d = 0.35). In the group of juveniles that have repeated a school year, the percentage of recidivism is 52.7%, while for the group of juveniles that have not repeated a year, this rate drops to 34.1%.

Next, we describe in detail the branch that is headed by the group of juveniles who do not relate with antisocial peers. Here the variable that determines the next split is the paternal parenting style (χ^2^_(1)_ = 20.68; p < .01; d = 0.50). In the group of juveniles whose father exercises little parental supervision, the percentage of recidivism is 32.2%, while for the group of juveniles whose father shows an adequate parenting style, this rate drops to 12.3%.

In the group of juveniles whose father exercises little parental supervision, the variable that determines the next split is mental health problems (χ^2^_(1)_ = 6.84; p < .01; d = 0.41); in the group of juveniles who present mental health problems, the percentage of recidivism is 57.1%, while for the group of juveniles do not present mental health problems, this rate drops to 28.7%.

The next split, dividing the juveniles that do not present mental health problems, is determined by the variable of juvenile’s age at the baseline incident (χ^2^_(1)_ = 4.42; p = .03; d = 0.35). The juveniles who were 16–17 years old present a percentage of recidivism of 21.7%, while juveniles who committed the baseline incident at age 14–15 show a rate of 37.7%.

Within the group of juveniles with adequate paternal parenting style, the variable that determines the next divide is school dropout (χ^2^_(1)_ = 18.08; p < .01; d = 0.65): the group of juveniles who had left school shows a level of recidivism of 25.4%, while for the group that continued their schooling this rate drops to 4.3%.

Within the group of juveniles who had left school, the final variable that prompts a split is having repeated a year in school (χ^2^_(1)_ = 10.85; p < .01; d = 0.85). In the group of juveniles who did not drop out of school, the final divide is whether the baseline incident was committed in the company of others (χ^2^_(1)_ = 5.56; p < .01; d = 0.45); in the group of juveniles who were accompanied in the offense, the percentage of recidivism is 1.3%, while for the group of juveniles who offended without the company of others, the percentage of recidivism rises to 10.8%.

## Discussion

The first objective of this study was to estimate the S-ASB recidivism rate of juveniles who had gone through the Juvenile Court de Almería. For this study, the recidivism rate is based on a new court case being opened in Almería province’s only Juvenile Court, during the two years following the juvenile’s baseline incident.

The rate of recidivism for this set of juveniles was estimated at 35.5%, similar to values obtained in other studies in Spain under Organic Law 5/2000 [17, 33,47,48]. When comparing educational measures applied to the young offender, the recidivism rate according to Capdevila et al. (2013) is 28.7% in cases of probation, and greater than 50% in cases of custodial sentences [17, 47]. The meta-analysis realized by Ortega, García and Frías (2014), using studies carried out under Organic Law 5/2000, estimates a recidivism rate of 26.89%, in line with other studies from different legislations [49–50].

Recidivism percentages are not directly comparable across studies from different countries [51], given that each country has its own law of penal responsibility for juveniles, the most important difference being the age at which juveniles are held legally responsible. Recidivism percentages among juveniles from different countries help us to understand the profile of the young offenders we are dealing with and whether the recidivism rate presents similar levels to those of other contexts. In this regard, we can affirm that the recidivism data found in this study are consistent with data from studies performed under legislation covering juveniles in other countries [18,46,52].

This study has presented a descriptive study of the individual, criminological and contextual variables of the total group of juveniles and of the group of recidivist juveniles. With respect to individual variables, the totality of juveniles from this study are largely male, with Spanish nationality [17,33,48,49,53]; they present deficiencies in the educational area [13,17–24]; and they lack organized leisure activities [17,18,20,28,29].

Regarding contextual variables, particularly noteworthy is antisocial peers [17,18,20,25–27], a lack of parental supervision [27,30], and criminality in family members [31,32].

We now proceed to a comparison of the results obtained from the total group of juveniles and from the group of recidivist juveniles. In the group of recidivist juveniles, the percentage of male youths is greater [33], they begin a path of delinquency at a younger age [18,33]; their educational problems are more severe (repeating a year in school, dropout) and they have less interest in their studies [13,17–24]. The group of recidivist juveniles presents greater substance abuse [54,55].

Regarding contextual variables, the group of recidivist juveniles shows higher levels of antisocial peers [17,18,20,25–27], problematic situations in home life and a lack of parental supervision [27,30].

Having studied the profiles represented by the total group of juveniles and the recidivist juveniles in particular, one may observe a large proportion of their characteristics are shared, with the recidivist juveniles presenting greater levels of the risk factors studied here. As indicated in PCC theory, facilitating or risk factors increase the probability of recidivism, while protection factors mitigate the effect of the risk factors [10]. This study confirmed that the recidivist juveniles present higher levels of characteristics associated with delinquency recidivism, particularly those factors that promote S-ASB as a way of life (antisocial peers and criminality in family members); by contrast, the recidivist juveniles present lower level of protection factors against S-ASB [13,14].

The second objective of this study was to further understand the profile of young offenders, particularly characteristics that differentiate the group of recidivist juveniles. Based on the decision tree that has been presented in this study, multiple variables show a relation to young offenders repeating S-ASB, and the variables that influence this are not limited to any single area, thereby reinforcing the theory that S-ASB is multi-causal and influenced by the different contexts of the juvenile [10,17,20,49].

In this study, the variables that show a relationship to repeated S-ASB, in order of the strength of their association, are as follows: antisocial peers [17,18,20,25–27]; age’s at the baseline S-ASB [18,33]; criminality in family members [31–32], school problems [17,18,20–24], father’s lack of parental supervision [27,30], the juvenile’s mental health [56–57] and being accompanied when committing the S-ASB.

According to the World Health Organization (2003) between 10–20% of the European adolescents suffer a mental health problem [58]. The Spanish Health Survey (2011–2012) estimated that a 2.6% of minors suffered behavioral disorder and 1.1% mental health problems. In 2013, the ANAR Foundation (ONG that help adolescents at risk) 11.11% of the calls to the help phone were related to children with psychological problems. In Spain, the White Book of Child and Adolescent Psychiatry (2014), indicate that 20% of minors may suffer a mental health problem.

The studies that analyzed the juvenile offenders under judicial measures showed percentages of mental health problems higher than 60%. However, when the studies were realized with the total number of juvenile offenders who were charged in a court case in the Juvenile Court [59], the percentage of younger offenders with mental health problems did not exceed the data of all the younger with mental health problems.

Regarding the national maltreatment rates, calculated for 100.000 minors, range from 98.1 in 2011, 111.7 in 2012 and 148.1 in 2013, in accordance with previous studies [60]. Other epidemiological studies were of very limited scope, due to the sampling method, as shown by the variety of the results.

In the present study, the data were extracted from the youth court records, the mental health should have been indicated in the younger forensic psychological assessment, otherwise it was considered that the young did not have any mental disorder. In this way, the data used for the study comprised the files of juveniles who were charged in a court case, not the youngers with a judicial measured. Therefore, this study should be used as reference data population of mental health problems, not data from sample of juvenile offenders with judicial measure.

Most of the more strongly predictive variables of S-ASB recidivism are factors indicated in the RNR model. The variable with the greatest predictive strength for recidivism is antisocial peers, a factor from *the big four*. The variables that make up the branch nodes in the decision tree are mostly found in *the moderate four*: family, school and free time and leisure [16]. Results obtained in national and international studies support the RNR model proposed by Andrews et al. (1990) [17,20].

Steadman et al. (2000) constructed a classification tree in order to identify groups of juveniles with high and low risk for violence, using the factors from the *MacArthur Violence Risk Assessment Study* [61] as predictive variables of violence. They identified profiles of juveniles using the results obtained in the decision tree. Following these authors’ approach, we grouped the juveniles in this study into levels of risk for recidivism. Our study grouped juveniles at three levels of risk for recidivism, high (>50%), medium (20–50%) and low (<20%), according to the percentage of risk for S-ASB recidivism that they present. Among the juveniles with a high risk of recidivism according to these criteria, we identified three groups (A, B and C). Group A, of which 80% were recidivist juveniles, contains juveniles who present antisocial peers, age 14–15 at the time of the baseline incident, dropout and a family history of delinquency. Group B presents a profile of antisocial peers, age 14–15 at the baseline incident and no dropout; their percentage of recidivism is about 60%. The difference between the two groups is in dropout [17–24], in the group that does not present this characteristic this acts as a mitigating factor of S-ASB recidivism. Group C presents recidivism about 50%, the juveniles relate with antisocial peers, they were 16–17 years old at the time of the baseline incident, and they have repeated a year in school.

According to the results obtained in the decision tree, the characteristics that trigger the highest delinquency recidivism in juveniles are antisocial peers, age at the baseline incident, dropout and family criminality. These factors share a common perspective of S-ASB as valid behavior. Juveniles with a high level of recidivism show S-ASB as accepted by friends and family, along with the age variable. Juvenile justice has the peculiarity of covering a limited age range; by establishing a two-year period in which to measure recidivism, some of the juveniles would have passed to the adult justice system, to which access was not available.

The medium level of risk for recidivism included juveniles whose risk percentage ranged from 20 to 50%; we identified 5 groups in this category. Group D, with a recidivism percentage about 30%, was made up of juveniles with antisocial peers, aged 16–17 at the baseline S-ASB and who had not repeated a year in school. In this group, not having repeated a year in school acts as a mitigating factor in recidivist conduct, while age at the baseline incident may mean that the juvenile leaves the juvenile justice system before the end of the two years defined for measuring recidivism.

Group E, with a percentage of recidivism of 21.7%, was formed by juveniles without antisocial peers, without adequate parental supervision from fathers, no mental health problems and age 16–17 at the time of the baseline incident. Group F presents the same factors as Group E, except for age at the baseline incident (14–15 years), which triggers a rise in the juveniles’ recidivism rate to 37.3%. In these groups, we confirm once again how age at the time of the baseline incident can act as a mitigating factor in S-ASB [33].

The other groups of juveniles with a medium level of recidivism risk are made up of juveniles who share the following characteristics: their peers are not antisocial, they present adequate parental supervision and they have dropped out of school. The differentiating factor between the two groups is having repeated a year in school: the juveniles who have dropped out of school and have repeated a school year present a lower level of recidivism, this fact may be due to the age of the juvenile. Having repeated a year in school and dropped out results in juveniles over the age of 16, which in turn leads to their exit from the juvenile justice system.

Finally, juveniles who present a low level of risk for S-ASB recidivism, with percentages between 1.3–10.8%, show the following: their peers are not antisocial, paternal parenting style is adequate, they have not dropped out of school. All these characteristics are prosocial and contrary to S-ASB as a way of life [13].

The results from the decision tree show the relationship between strengthening and mitigating factors in predicting S-ASB recividism. Juveniles who present a greater number of risk factors and the absence of protection factors present higher levels of recidivism, while juveniles who present an absence of risk factors and the presence of protection factors refrain from repeating S-ASB [10–12].

The Juvenile Justice investigation is focused in the study of a group of younger offenders taking into account the characteristics of the younger (eg: sex, nationality, health problems, etc.) [62–64] or the S-ASB committed (eg: type of S-ASB, type of judicial measure, etc) [65–66]. This type of study present the advantage that allows to have an extensive knowledge of youngers with this characteristics, but not about all the juvenile offenders who go through the system of Juvenile Justice.

Once the younger had gone through a judicial measure, his characteristics have been influenced by the program in which he had taken part such as internament measures. For this reason, the characteristics as *mental health problems* may present higher values in comparison with adolescents as a consequence of the measures taken with the younger offender, measures that can have a positive or negative result.

The objective of the present study was to estimate the characteristics of juvenile offenders that passed through Juvenile Justice in order to help in the planning of preventing or rehabilitation programs. One of the novelties of this study is the use of the younger offender characteristics in the moment of the arrest. The variables of the younger in the moment of the arrest should be taken into account as a reference point for the establishment of the prevention/rehabilitation programs [67].

In the present study a multivariate approximation of the variables that predict the juvenile offenders recidivism is presented because when the univariate analysis is carried out, the information about the interaction effects between the variables is lost. Therefore, the answer tree analysis presents the advantage of studying the effect of the variables together, ordering them according to the strength of association with the younger offenders recidivism. In addition, the graphical representation of the results is easy to be interpreted by justice system workers that are not experts in research, but that should know what are the variables that present a higher strength of association in relation with the recidividism of the younger offenders to be able to apply properly the prevention programs.
